# New regression equations for mixed dentition space analysis in Nepalese mongoloids

**DOI:** 10.1186/s12903-018-0677-1

**Published:** 2018-12-13

**Authors:** Jamal Giri, Prabhat Ranjan Pokharel, Rajesh Gyawali, Jigyasha Timsina, Kashmira Pokhrel

**Affiliations:** Department of Orthodontics, B.P. Koirala Institute of Health Sciences, Dharan, Nepal

**Keywords:** Mixed dentition space analysis, Moyers’ analysis, Nepalese mongoloids, Regression equations, Tanaka-Johnston analysis

## Abstract

**Background:**

Mixed dentition space analysis methods using regression equations, namely, Moyers’ analysis and Tanaka-Johnston analysis are commonly used around the world. However, the applicability of these analyses among different racial groups have been questioned. The primary objective of this study was to assess the applicability of the Moyers’ and Tanaka-Johnston analyses among Nepalese Mongoloids and to develop regression equations for the same population if needed.

**Methods:**

One hundred (50 males and 50 females) pre-treatment study models of the Nepalese Mongoloid patients undergoing orthodontic treatment were retrieved from the archives of the department of Orthodontics. The mesiodistal widths of mandibular incisors and widths of canines and premolars of all 4 quadrants were measured by a single investigator using a digital caliper to the nearest 0.01 mm. Predicted widths of canines and premolars were obtained using standard Moyers’ and Tanaka-Johnston analyses and then compared with the measured widths.

**Results:**

The measured widths of canines and premolars were significantly different from the predicted widths obtained from Moyers’ and Tanaka-Johnston analyses. Strong and positive correlations were found between the sum of mesiodistal widths of mandibular incisors and the sum of mesiodistal widths of canines and premolars in males (0.73 for maxillary arch and 0.68 for mandibular arch) and females (0.64 for maxillary arch and 0.79 for mandibular arch).

**Conclusions:**

The Moyers’ and Tanaka-Johnston analyses did not accurately predict the mesiodistal width of unerupted canines and premolars for Nepalese Mongoloid population. Hence, new regression equations have been developed for this population. However, validation studies should be conducted to confirm the applicability and accuracy of these equations.

**Electronic supplementary material:**

The online version of this article (10.1186/s12903-018-0677-1) contains supplementary material, which is available to authorized users.

## Introduction

When the mandibular first permanent molars or incisors erupt in the oral cavity at around 6 years of age, mixed dentition stage begins. It is a transitional stage between the deciduous and permanent dentition which lasts till all the deciduous teeth have been replaced by permanent teeth. From orthodontic point of view, it is an important period of occlusal development because most of the developing malocclusions become apparent at this stage. Interception of developing malocclusion in the mixed dentition stage can reduce the severity or totally eliminate the malocclusion in future [1]. However, any interception at this stage should be preceded by a mixed dentition space analysis [2, 3].

Mixed dentition space analysis is a method of predicting the mesiodistal widths of unerupted permanent teeth: canine, first and second premolar. The prediction of mesiodistal widths of unerupted canines and premolars can be done by radiographic or non-radiographic approach [4]. No matter which approach is used, this analysis helps clinician predict the possible crowding or spacing in each quadrant of the oral cavity. Accordingly, the clinician can opt for serial extraction, space maintenance, space regaining or just regular monitoring of the patient [5, 6]. Prediction of width of permanent canines and premolars can be done as early as in primary dentition using Boston University approach but the clinical implication of this prediction is undermined by the changes in arch dimensions, tooth position and inclination. [7]

Moyers’ analysis [8] and Tanaka-Johnston analysis [9] are commonly used mixed dentition analyses around the globe. However, studies have shown that these analyses which were developed for North European and North American population might not be accurate for other populations of different ethnicities [10–16]. Till date, two studies assessing the applicability of Moyers’ and Tanaka-Johnston analyses among Nepalese samples have been published and both studies have concluded that the standard Moyers’ and Tanaka-Johnston analyses do not accurately predict the widths of unerupted teeth in both the arches for Nepalese population [17, 18]. Based on these studies two types of mixed dentition prediction equations are available for Nepalese population, namely, Jaiswal’s equations [17] where the ethnicity is not specified and Gyawali’s equations [18] for Nepalese Brahmins/chhetris.

Nepal is a multi-ethnic country with Mongoloids comprising one-fifth of the total population. But, data regarding the mixed dentition space analysis among Nepalese Mongoloids is still lacking. Hence, the primary objective of this study was to assess the applicability of the Moyers’ and Tanaka-Johnston analyses among Nepalese Mongoloids and to develop prediction equations for the same population if needed. The secondary objective was to assess the applicability of Jaiswal’s and Gyawali’s prediction equations for Nepalese Mongoloids. The null hypothesis of the study was that the Moyers’ analysis and Tanaka-Johnston analysis accurately predict the mesiodistal widths of unerupted canines and premolars of Nepalese Mongoloid population.

## Methods

This cross-sectional study was conducted at the department of Orthodontics, BP Koirala Institute of Health Sciences (BPKIHS), Dharan, Nepal. Ethical approval of the study was obtained from the institutional review board of BPKIHS (IRC/0829/016). One hundred (50 males and 50 females) pre-treatment study models of the patients undergoing orthodontic treatment were retrieved from the archives of the department. Patients’ record files were used to determine their ethnic origin. The samples were selected using non-probability convenient sampling technique based on the following criteria.

Inclusion criteria

1. Both parents and grandparents Nepalese Mongoloids
2. All permanent teeth till the first molar fully erupted

Exclusion criteria

1. History of orthodontic treatment
2. Interproximal caries or restoration
3. Macrodontia or microdontia
4. Attrition, abrasion, erosion or gross dental abnormalities

For sample size calculation, the value of standardized difference (0.67) of a previous study was used [18].

$$ \mathrm{Standardized}\ \mathrm{difference}=\frac{clinically\ important\ smallest\ difference}{standard\ devi\mathrm{a} tion\ of\ the\ difference}=\frac{0.2}{0.3}=0.67 $$

Also, the following assumptions were made.

- Power of the test = 0.8
- Significance level = 0.05
- Confidence level = 95%

To achieve a power of 0.8 at a significance level of 0.05 with standardized difference of 0.67, 100 samples were needed according to Altman Nomogram [19]. Therefore, 100 study models (50 males and 50 females) were selected for this study.

A single investigator (JG) measured all the study models using a digital vernier caliper (Mitutoyo: CD-8” CS, Japan) with accuracy of 0.01 mm in natural light. The mesiodistal crown widths were recorded by measuring the maximum distance between the contact points on proximal surfaces of teeth. The caliper was held parallel to the occlusal surface and perpendicular to the tooth’s long axis during all measurements. The method described by Hunter and Priest was followed during the measurement to minimize errors [20]. The mesiodistal widths of mandibular incisors were measured followed by the measurement of widths of canine, first and second premolars of all 4 quadrants. To minimize the investigator fatigue only 5 study models were measured in one sitting and the number of sittings were limited to 2 per day. Twenty five percent of the study models were re-measured 2 weeks after the initial measurement to assess intra-examiner reliability.

Data was entered in Microsoft office excel sheet (Office 10) and transferred to SPSS software (version 11.5) for statistical analyses. Kolmogorov-Smirnov test was used to assess the normality of the data. Pertinent descriptive statistics like mean, standard deviation and standard error of mean were calculated. An independent sample t-test was performed to assess any significant difference: (i) between mesiodistal widths of teeth in male and female samples and (ii) between mesiodistal widths of teeth of right and left side of the arch. A paired sample t-test was used to compare the means of measured and predicted mesiodistal widths of permanent canines and premolars according to Moyers’ and Tanaka Johston analysis. A bivariate correlation (Pearson’s correlation) analysis was used to evaluate the relationship between the independent variable (sum of mesiodistal widths of mandibular incisors) and dependent variable (sum of mesiodistal width of canine and premolars of maxillary and mandibular arches). Finally, linear regression models for predicting the sum of mesiodistal widths of canines and premolars of both arches from the sum of mesiodistal widths of mandibular incisors were derived using simple linear regression analysis. One way ANOVA was used to compare the means of predicted sum of mesiodistal widths of canines and premolars according to equations derived in this study, Jaiswal’s equations and Gyawali’s equations. The level of significance was set at 5%.

## Results

The samples (50 males and 50 females) had a mean age of 18.6 ± 3.87 years and 18.76 ± 4.01 years for males and females respectively. The data (mesiodistal widths of mandibular incisors, canines and premolars) was found to be normally distributed when subjected to Kolmogorov Smirnov test (*p* > 0.05). Intraclass correlation coefficient (ICC) with absolute agreement was calculated to determine the intra-examiner reliability which suggested excellent agreement (ICC = 0.91).

When the sum of mesiodistal widths of canines and premolars of the right and the left side of the dental arch were compared, there were no statistically significant differences between the two sides in both maxillary and mandibular arches (*p* > 0.05). Hence, the sum of mesiodistal widths of canines and premolars of the right and left side were averaged for further analysis. However, there were statistically significant differences in the sum of mesiodistal widths of mandibular incisors and sum of mesiodistal widths of canines and premolars between male and female samples (*p* < 0.05, t test; Table 1).

**Table 1:** Sum of mesiodistal widths of mandibular incisors, maxillary and mandibular canines and premolars (mm) according to gender

Group of teeth	Gender	N	Mean	S.D	S.E.M	*p*-value
Mandibular incisors	Male	50	23.22	1.60	0.23	0.016
	Female	50	22.45	1.53	0.22	
Maxillary canines and premolars	Male	50	22.54	1.11	0.16	< 0.01
	Female	50	21.45	1.26	0.18	
Mandibular canines and premolars	Male	50	21.63	1.11	0.16	< 0.01
	Female	50	20.62	1.18	0.17	

*N*: Number of samples; *S.D*.: Standard deviation; *S.E.M*: Standard error of mean

Moyers’ probability table and Tanaka-Johnston equations were applied to the study samples to compare between the predicted sum of mesiodistal widths of canines and premolars and the measured sum of mesiodistal widths of the teeth. T-test revealed statistically significant differences between the predicted and measured sum of the teeth (canines and premolars) in both maxillary and mandibular arches for male and female samples (Table 2 and Table 3).

**Table 2:** Comparison of measured and predicted sum of mesiodistal widths of canines and premolars according to Moyers’ method

Gender	Arch	Probability %	Mean Difference	S.D	S.E.M	95% CI		t	*p*-value
						Lower	Upper		
Male	Maxilla	75	0.43	0.76	0.11	0.21	0.64	3.98	0.001
		50	0.96	0.76	0.11	0.75	1.18	8.98	0.001
	Mandible	75	−0.39	0.81	0.11	−0.62	− 0.16	−3.39	0.001
		50	0.45	0.81	0.11	0.22	0.68	3.93	0.001
Female	Maxilla	75	0.26	1.04	0.15	−0.04	0.56	1.77	0.083
		50	0.96	1.04	0.15	0.66	1.26	6.52	0.001
	Mandible	75	−0.45	0.72	0.10	−0.66	− 0.25	− 4.42	0.001
		50	0.35	0.72	0.10	0.14	0.55	3.39	0.001

*S.D*: Standard deviation; *S.E.M*: Standard error of mean

**Table 3:** Comparison of measured and predicted sum of mesiodistal widths of canines and premolars according to Tanaka-Johnston method

Gender	Arch	Mean difference	S.D	S.E.M	95% CI		t	*p*-value
					Lower	Upper		
Male	Maxilla	−0.78	0.97	0.14	−1.05	−0.50	−5.69	< 0.001
	Mandible	−1.10	0.73	0.10	−1.31	−0.90	−10.68	< 0.001
Female	Maxilla	−0.43	0.93	0.09	−0.61	−0.24	−4.56	< 0.001
	Mandible	−0.79	0.83	0.08	−0.96	−0.63	−9.58	< 0.001

*S.D*: Standard deviation; *S.E.M*: Standard error of mean

Sum of mesiodistal widths of mandibular incisors of the study samples displayed strong and positive correlations with sum of mesiodistal widths of canines and premolars of maxillary and mandibular arches. Hence, linear regression models for predicting the sum of mesiodistal widths of canines and premolars of both arches from the sum of mesiodistal widths of mandibular incisors were derived (Table 4). By substituting the values of constants a and b (from Table 4) in the eq. Y = a + bx, four regression equations were formulated. (Table 5).

**Table 4:** Regression characteristics

Gender	Arch	r	r^2^	Regression coefficient		S.E.E	95% CI		*p*-value
				a	b		Lower	Upper	
Male	Maxilla	0.73	0.53	10.72	0.51	0.76	0.37	0.64	< 0.001
	Mandible	0.68	0.47	10.56	0.47	0.81	0.33	0.62	< 0.001
Female	Maxilla	0.64	0.41	9.51	0.53	0.97	0.34	0.71	< 0.001
	Mandible	0.79	0.63	6.87	0.61	0.71	0.47	0.74	< 0.001

r: Correlation coefficient; r^2^: Coefficient of determination; a and b: Linear regression constants; *S.E.E*: Standard error of estimate

**Table 5:** Regression equations for Nepalese Mongoloids

Gender	Arch	Equation
Male	Maxilla	Y = 10.72 + 0.51x
	Mandible	Y = 10.56 + 0.47x
Female	Maxilla	Y = 9.51 + 0.53x
	Mandible	Y = 6.87 + 0.61x

x: sum of mesiodistal width of mandibular incisors; Y: Sum of mesiodistal width of canine, first and second premolars

A prediction table for the most commonly encountered sum of mesiodistal widths of mandibular incisors was constructed using the regression equations (Table 6).

**Table 6:** Prediction table for Nepalese Mongoloids

Σ MI (mm)	Male		Female	
	Σ CP1P2 (mm)	Σ CP1P2 (mm)	Σ CP1P2 (mm)	Σ CP1P2 (mm)
	Maxilla	Mandible	Maxilla	Mandible
18	19.9	19.1	19.1	17.9
18.5	20.1	19.4	19.3	18.2
19	20.4	19.6	19.6	18.5
19.5	20.6	19.9	19.9	18.8
20	20.9	20.1	20.1	19.1
20.5	21.2	20.3	20.4	19.4
21	21.4	20.6	20.7	19.7
21.5	21.7	20.8	20.9	20.0
22	21.9	21.0	21.2	20.3
22.5	22.2	21.3	21.5	20.7
23	22.4	21.5	21.7	21.0
23.5	22.7	21.8	22.0	21.3
24	22.9	22.0	22.3	21.6
24.5	23.2	22.2	22.5	21.9
25	23.4	22.5	22.8	22.2
25.5	23.7	22.7	23.1	22.5
26	24.0	23.0	23.3	22.8
26.5	24.2	23.2	23.6	23.1
27	24.5	23.4	23.9	23.4
27.5	24.7	23.7	24.1	23.7
28	25.0	23.9	24.4	24.0
28.5	25.2	24.1	24.7	24.3
29	25.5	24.4	24.9	24.6

*Σ MI*: Sum of mesiodistal widths of mandibular incisors; *Σ CP1P2*: Sum of mesiodistal widths of canines and premolars

One way ANOVA was used to compare the means of predicted sum of mesiodistal widths of canines and premolars according to equations derived in this study, Jaiswal’s equations and Gyawali’s equations. There were statistically significant differences between the groups in the maxillary (F = 16.75, *p* < 0.001) and mandibular (F = 7.59, p < 0.001) arches among male samples only. A Tukey post hoc test revealed statistically significant differences in predicted sum of mesiodistal widths of canines and premolars between: 1) this study’s equations and Jaiswal’s equations in both maxillary (mean difference = 0.98 mm, p < 0.001) and mandibular (mean difference = 0.69 mm, p < 0.001) arches among male samples and 2) this study’s equations and Gyawali’s equations in maxillary arch (mean difference = 0.62 mm, p < 0.001) among male samples (Additional file 1).

## Discussion

Accurate prediction of mesiodistal widths of unerupted permanent canines and premolars during the mixed dentition stage is of paramount importance for the practice of preventive and interceptive orthodontics. But, mesiodistal widths of permanent teeth vary according to the ethnicity of a population, Nepalese Mongoloid population is no exception. Two most commonly used methods (Moyers’ analysis and Tanaka-Johnston analysis) to predict the mesiodistal widths of unerupted permanent canines and premolars were developed for North European and North American population, hence, might not accurately predict teeth dimensions of Nepalese Mongoloid population. Therefore, this cross-sectional study was conducted to assess the applicability of the Moyers’ and Tanaka-Johnston analyses among Nepalese Mongoloids and to develop prediction equations for the same population if needed.

Our study revealed that the Tanaka-Johnston method of prediction overestimates the mesiodistal widths of unerupted permanent canines and premolars of Nepalese Mongoloids in maxillary and mandibular arches for both genders. This finding is in agreement with those obtained by Lee-Chan et al. [21], Diagne et al. [22], Al-Bitar et al. [23] and Buwembo et al. [24]. However, studies done in Thai [15] and Kenyan [25] population have shown that the Tanaka-Johnston method is an accurate method of tooth dimension prediction. Our study also demonstrated that there were statistically significant differences between the measured and predicted widths of permanent canines and premolars obtained using Moyers’s table except at 75th percentile in maxilla for females. Since, Moyers [8] recommends using the 75th or 50th percentile level of probability, the Moyers table is only applicable for predicting the mesiodistal widths of maxillary canines and premolars of Nepalese Mongoloid females. This lack of applicability of Tanaka-Johnston and Moyers’s equations for Nepalese Mongoloid population can be attributed to racial and ethnic variations.

Studies have shown that the sum of mesiodistal widths of permanent mandibular incisors is the best predictor of the sum of unerupted permanent canines and premolars. [26, 27] Therefore, we planned to develop two regression equations (one each for maxilla and mandible) to predict the sum of mesiodistal widths of unerupted permanent canines and premolars using the sum of mesiodistal widths of permanent mandibular incisors as predictor variable. However, sexual dimorphism in the mesiodistal dimension of the permanent teeth was observed in our study with the mesiodistal dimension of male teeth being greater than their female counterparts. Several studies have reported similar finding. [28, 29] Therefore, separate equations were developed for males and females for maxillary and mandibular arches. But, there was no statistically significant difference between the mesiodistal width of teeth of right and left side of the jaws for both genders. Hence, the mesiodistal widths of teeth of the right and the left sides were averaged for further calculations.

In our study, correlation coefficients (r) between the sum of mesiodistal widths of permanent mandibular incisors and the sum of mesiodistal width of maxillary and mandibular canines and premolars ranged between 0.64 and 0.79 suggestive of strong and positive correlations. These strong correlations enable accurate prediction of the sum of mesiodistal widths of unerupted canines and premolars when the sum of mesiodistal widths of permanent mandibular incisors is known. The correlation coefficient values of our study were comparable to that of Nepalese Brahmins/Chhetris [18] and Hong Kong Chinese [11] but higher when compared to Jordanian [23], Thai [15] and Syrian [16] populations. However, there are studies which have reported higher correlation coefficient values than our study. [24, 30]

Coefficient of determination (r^2^) in our study ranged between 0.41 and 0.63 suggesting that 41 to 63% of the total variation in the sum of mesiodistal widths of unerupted canine and premolars (y) can be explained by the sum of mesiodistal widths of mandibular incisors (x). The r^2^ values of our study were comparable to that of Nepalese Brahmins/Chhetris [18] and Hong Kong Chinese [11] but higher when compared to Han Chinese [12], Saudi Arabian [31], Senegalese [22], Thai [15] and Syrian [16] population. However, studies conducted in Turkey [30] and Uganda [24] have reported higher r^2^ values than our study.

Standard error of the estimate (SEE) measures the accuracy of prediction equation; the lower the SEE, the more accurate the prediction equation. The SEE values of our equations ranged between 0.71 and 0.97 which are similar to Thai [15], Syrian [16], Senegalese [22], Pakistani [10], Black American [32] population but more than Turkish [30] population.

Jaiswal et al. [17] found that the Moyers’ analysis underestimated and the Tanaka-Johnston analysis overestimated the sum of mesiodistal widths of unerupted permanent canines and premolars of Nepalese population. Even though it was a first study of its kind conducted among Nepalese population it did not consider the ethnic variation prevalent in Nepal. Since, Nepal is a multiethnic country, ethnic variations should be given due consideration while developing regression equations for mixed dentition space analysis among Nepalese population. When the sum of mesiodistal widths of permanent canines and premolars of our study were compared with the predicted widths given by Jaiswal et al., statistically significant differences were found in both maxillary and mandibular arches in male samples only. Based on this finding, it might seem reasonable to use Jaiswal’s equations for Nepalese Mongoloid females. However, this finding should be interpreted with caution. Ambiguity over the ethnicity of the samples selected means that the Jaiswal’s equations have limited clinical use.

When the sum of mesiodistal widths of permanent canines and premolars of our study were compared with the predicted width given by Gyawali et al. [18], there were no statistically significant differences except for the sum of mesiodistal widths of maxillary permanent canines and premolars of male samples. It can thus be suggested that the regression equations developed for Nepalese Mongoloid females can also be used for Nepalese Brahmin/Chhetris females. This is an interesting finding and hence needs further investigation.

Our study has developed regression equations for predicting the sum of mesiodistal widths of unerupted canines and premolars for Nepalese Mongoloids which need to be validated by further studies. Validation studies to determine the applicability and prediction accuracy of the regression equations are therefore recommended. Few studies have suggested that the prediction accuracy of regression equation can be improved by selecting samples without intermaxillary tooth size discrepancy. [30, 33] This is an important issue for future research.

## Conclusions

- The Moyers’ analysis and Tanaka-Johnston analysis are not suitable for accurate prediction of mesiodistal widths of unerupted canines and premolars of Nepalese Mongoloid population.
- The following prediction equations have been developed for Nepalese Mongoloids from this study:

Nepalese Mongoloid males

- Maxilla: y = 10.72 + 0.51x
- Mandible: y = 10.56 + 0.47x

Nepalese Mongoloid females

- Maxilla: y = 9.51 + 0.53x
- Mandible: y = 6.87 + 0.61x
- this study:

- Validation studies in a similar population, preferably with a larger sample size, should be conducted to confirm the applicability and accuracy of these regression equations.

## Additional file

Additional file 1: Master table of the data used in the research. (SAV 30 kb)
